# FlyPrimerBank: An Online Database for *Drosophila melanogaster* Gene Expression Analysis and Knockdown Evaluation of RNAi Reagents

**DOI:** 10.1534/g3.113.007021

**Published:** 2013-09-01

**Authors:** Yanhui Hu, Richelle Sopko, Marianna Foos, Colleen Kelley, Ian Flockhart, Noemie Ammeux, Xiaowei Wang, Lizabeth Perkins, Norbert Perrimon, Stephanie E. Mohr

**Affiliations:** * Department of Genetics, Harvard Medical School, Boston, Massachusetts 02115; † *Drosophila* RNAi Screening Center, Department of Genetics, Harvard Medical School, Boston, Massachusetts 02115; ‡ Departments of Radiation Oncology, Washington University School of Medicine, St. Louis, Missouri 63108; § Howard Hughes Medical Institute, Boston, Massachusetts 02115

**Keywords:** *Drosophila*, real-time PCR, gene expression, RNAi, knockdown evaluation

## Abstract

The evaluation of specific endogenous transcript levels is important for understanding transcriptional regulation. More specifically, it is useful for independent confirmation of results obtained by the use of microarray analysis or RNA-seq and for evaluating RNA interference (RNAi)-mediated gene knockdown. Designing specific and effective primers for high-quality, moderate-throughput evaluation of transcript levels, *i.e.*, quantitative, real-time PCR (qPCR), is nontrivial. To meet community needs, predefined qPCR primer pairs for mammalian genes have been designed and sequences made available, *e.g.*, via PrimerBank. In this work, we adapted and refined the algorithms used for the mammalian PrimerBank to design 45,417 primer pairs for 13,860 *Drosophila melanogaster* genes, with three or more primer pairs per gene. We experimentally validated primer pairs for ~300 randomly selected genes expressed in early *Drosophila* embryos, using SYBR Green-based qPCR and sequence analysis of products derived from conventional PCR. All relevant information, including primer sequences, isoform specificity, spatial transcript targeting, and any available validation results and/or user feedback, is available from an online database (www.flyrnai.org/flyprimerbank). At FlyPrimerBank, researchers can retrieve primer information for fly genes either one gene at a time or in batch mode. Importantly, we included the overlap of each predicted amplified sequence with RNAi reagents from several public resources, making it possible for researchers to choose primers suitable for knockdown evaluation of RNAi reagents (*i.e.*, to avoid amplification of the RNAi reagent itself). We demonstrate the utility of this resource for validation of RNAi reagents *in vivo*.

Quantitative, real-time PCR (qPCR) is widely used for analysis of transcript levels because it is sensitive, accurate, relatively easy to perform, and can be adapted to moderately high-throughput modes. qPCR has become important for the study of transcriptional regulation, for example, to detect changes in specific transcript levels after treatment with different stimuli. In particular, qPCR is commonly used to confirm results obtained using microarray analysis, methods enabling the detection and quantitation of multiple RNAs such as Nanostring (Geiss *et al.* 2008) and Luminex (Peck *et al.* 2006), or RNA-seq, as well as to evaluate the knockdown efficiency of RNA interference (RNAi) reagents. One barrier to performing qPCR assays efficiently is that the design of oligonucleotide primers for qPCR is not as straightforward as the design of primers for conventional PCR or sequencing because qPCR is much more sensitive to nonspecific amplification (Wang and Seed 2003). Several tools and resources have been developed to assist people in designing qPCR primers, including qPrimerDepot, PrimerBank and RTPrimerDB (Cui *et al.* 2007; Lefever *et al.* 2009; Pattyn *et al.* 2003, 2006; Spandidos *et al.* 2010; Wang and Seed 2003; Wang *et al.* 2012). qPrimerDepot provides qPCR primer sequences for 99.96% of human RefSeq sequences (Cui *et al.* 2007), and PrimerBank is an online genome-scale primer resource for human and mouse genes (Spandidos *et al.* 2010; Wang and Seed 2003; Wang *et al.* 2012). RTPrimerDB is a collection of 8609 experimentally validated qPCR primer sequences from the scientific community for 27 different species but currently only 5 primer pairs in RTPrimerDB target *Drosophila* genes (Lefever *et al.* 2009; Pattyn *et al.* 2003, 2006). Relevant to our goals, none of these resources provides comprehensive coverage of *Drosophila* genes.

RNAi is a widely adopted experimental tool for loss-of-function studies. Unlike the siRNA reagents used in mammalian systems, long double-stranded RNA (dsRNA) sequences of approximately 200−500 bps are common in reagent libraries for *Drosophila* cell-based RNAi. For *Drosophila in vivo* studies, RNAi reagents are generally either long dsRNA hairpins (usually between 200 and 500 bps) with gene fragments cloned by PCR as inverted repeats, or short hairpins (shRNAs) of 19−21 bps generated from oligonucleotides (Clemens *et al.* 2000; Hammond *et al.* 2000). Genome-scale RNAi reagents targeting *Drosophila* genes have been made available by several independent groups (Dietzl *et al.* 2007; Flockhart *et al.* 2011; Horn *et al.* 2010; Ni *et al.* 2009, 2011; Perrimon *et al.* 2010; Yamamoto 2010). Studies using RNAi reagents in cultured cells, as well as *in vivo* in *Drosophila*, have made contributions to a number of areas of study, and qPCR analysis is a common method used to assess the level of target gene knockdown. To accurately assess dsRNA-mediated knockdown, qPCR primer pairs must not amplify regions that are also part of the reagent sequence; otherwise, the primers might amplify the RNAi reagent itself. This is not a concern for shRNA reagents, which are generally too small to be amplified by qPCR primers.

Thus, although several qPCR primer design tools are available, there remains a need for a comprehensive and quality-analyzed set of primers useful for *Drosophila*, including for the analysis of RNAi-mediated knockdown. To address this need, we implemented the PrimerBank algorithm for *Drosophila* genes and designed three or more primer pairs for each *Drosophila* protein-coding gene. We supplemented the resource using an alternative algorithm with additional primer pairs for genes for which the PrimerBank design algorithm generated fewer than 3 primer pairs per gene. After primer design, we systematically evaluated the overlap of each primer with long dsRNA reagents for *Drosophila* genes from publicly available sources. A subset of the FlyPrimerBank primers were experimentally evaluated with the use of SYBR Green-based thermal analysis as well as gel electrophoresis and sequencing of PCR products after conventional PCR with 326 randomly selected *Drosophila-*specific primer pairs. In addition, we assessed primers in two additional test cases. One was the evaluation of a collection of transgenic fly lines bearing shRNAs for RNAi to knock down protein kinases and phosphatases. The other was the stimulation of the Jun N-terminal kinase (JNK) pathway in *Drosophila* S2 cells. Finally, we have made FlyPrimerBank available online, including an option for user input and feedback, making FlyPrimerBank a useful community resource that can further improve over time.

## Materials and Methods

### Primer design and annotation

Sequence information for *Drosophila* genes was retrieved from FlyBase (ftp://ftp.flybase.net/releases/). Coding DNA sequences (CDS) were formatted as input for uPrimer, the primer design program implemented in Perl for PrimerBank. Up to three primer pairs per gene were selected based on primer parameters as well as predicted gene specificity (Wang and Seed 2003). The alternative algorithm and primer annotation program was developed in Java. *Drosophila* RNAi Screening Center at Harvard Medical School (DRSC) and TRiP RNAi reagent information were retrieved from flyrnai.org. National Institute of Genetics at Japan (NIG) stock information was retrieved from the NIG catalog (http://www.shigen.nig.ac.jp/fly/nigfly/download/files/rnai.tsv). Vienna Drosophila RNAi Center (VDRC) stock information was retrieved from the VDRC catalog (http://stockcenter.vdrc.at/control/fullCatalogueExcel). The primer sequences for making double-stranded long RNAi reagents were collected and amplicon sequences of RNAi reagents were assembled by virtual PCR based on FlyBase release 5.51 using UP-TORR (Hu *et al.* 2013). The primer sequences from FlyPrimerBank were blasted against the sequences of RNAi reagents and the overlap of each primer sequence with RNAi reagents was analyzed using a program developed in-house in Java.

### Isolation of embryonic RNA

Approximately 300 embryos (0- to 4-hr old) were collected and chorions were removed by incubation for 5 min in 50% bleach. Embryos were washed with 0.1% TritonX-100, then an equal volume of Trizol (Invitrogen) and RNase-free 0.5-mm glass beads (Next Advance) were added to an Eppendorf Safe-Lock 1.5-mL microcentrifuge tube (VWR). Embryos were homogenized by bead beating 3 × 3 min at 4° at a setting of 8 in a Bullet Blender (Next Advance) and stored at −80° until further processing. RNA was extracted with chloroform and precipitated with isopropanol. An RNA pellet was re-suspended in RDD buffer (QIAGEN) and incubated with DNAse I (QIAGEN) for 10 min at room temperature. The sample was then diluted in RLT buffer and ethanol. Further cleanup proceeded with an RNeasy MinElute Cleanup Kit (QIAGEN). RNA was eluted with RNAse-free water. RNA concentration and purity (criteria: A_260_/A_280_ ratio near 2) was assessed using a Nanodrop 8000 spectrophotometer (Thermo-Scientific).

### Generation of embryonic cDNA

A total of 1 μg of RNA was incubated with a mix of oligo(dT) and random hexamer primers in iScript reaction mix and iScript reverse transcriptase (iScript cDNA Synthesis Kit; Bio-Rad) for reverse transcription. Reaction conditions were: 5 min at 25°, 30 min at 42°, and 5 min at 85°.

### Evaluation of primers by thermal analysis/calibration curve analysis

cDNA from embryos expressing a control shRNA targeting EGFP was serially diluted four times by a factor of four (Bio-Rad), starting with 1/20th of cDNA synthesis reaction volume. qPCRs included each primer at 0.4 μM in iQ SYBR Green Supermix with a reaction volume of 13 μL. R-squared values and PCR efficiency were calculated using Bio-Rad CFX Manager based on the results of a two-step qPCR program (40 cycles, alternating between 10 sec at 95° and 30 sec at 56°) using a Bio-Rad CFX96 Touch Real-Time PCR Detection System. Melt curve analysis was based on temperature ramping from 55° to 95° in 0.5° increments over 5 min.

### Evaluation of primers by conventional PCR

PCR master mix included 15 microliters 2x GoTaq Green (Promega), 11.65 μL of water, 2.5 μL of cDNA and 0.85 μL of primer (10 μM forward and reverse). The PCR program was 95°, 2 min, then 40 cycles alternating between 30 sec at 95°, 30 sec at 60°, and 40 sec at 72°. Four microliters of PCR product was run on a 1% agarose gel for visual inspection of size, and 26 μL of the remaining PCR product was transferred to a flat bottom 96-well PCR plate (VWR). Ninety microliters of PM buffer (QIAquick 96-well PCR Purification kit; QIAGEN), were added to each sample, mixed by pipetting, and transferred to the QIAquick PCR purification column plate. Once added, the samples were vacuumed to remove the buffer and washed with 900 μL of PE buffer. The purification column plate was spun at 4000 rpm for 5 min and vacuumed for 5 min to remove ethanol. The column plate was placed into a Nunclon 96-well plate (Sigma-Aldrich). Sixty microliters of water were added to each well, incubated at room temperate for 2 min, and spun at 3220 g for 5 min. The concentration of the purified DNA was measured using a Nanodrop 8000 spectrophotometer (Thermo-Scientific), which ranged from 5 to 45 ng/μL. Purified DNA samples were sent for Sanger sequencing (Dana Farber/Harvard Cancer Center DNA Resource Core) using a sequencing primer downstream of the forward PCR primer. Sequencing results were aligned to the *Drosophila* transcriptome using NCBI Blast.

### Assessment of transcript knockdown in shRNA-expressing embryos

Expression of shRNAs targeting EGFP (control) and shRNAs targeting various PKs was induced specifically in the female germline using the Gal4-UAS system (Brand and Perrimon 1993). Females heterozygous for the UAS-shRNA and either MTD-Gal4 (Petrella *et al.* 2007), a line bearing three versions of Gal4 expressed sequentially throughout oogenesis, or tub-Gal4, a line bearing two insertions of Gal4 expressed from a maternal tubulin promoter during mid and late oogenesis (Staller *et al.* 2013), were crossed to shRNA-bearing males to recover fertilized eggs. RNA was prepared as indicated previously, from 0- to 4–hr-old eggs derived from Gal4/shRNA females cultured at 27°. cDNA was generated from 1 μg of purified RNA as outlined previously. qPCR analysis was performed twice with technical triplicates using validated primers (above) in iQ SYBR Green Supermix (Bio-Rad), using a CFX96 Real-Time PCR detection system (Bio-Rad). Query transcript detection was normalized to the expression of three reference genes: *RpL32*, *alphaTub84B*, and either *nuclear fallout* or *Gapdh1*. These reference genes range in expression from high to low in 0-4 hr embryos, based on RNA-Seq data (Graveley *et al.* 2011).

### Monitoring stimulation of the JNK pathway in cells

The lipopolysaccharide (LPS)-responsive Schneider S2 cells were grown at 25° in Schneider’s *Drosophila* medium (GibcoBRL) supplemented with 10% fetal bovine serum, and antibiotics (50 units/mL penicillin and 50 μg/mL streptomycin). A commercial LPS preparation (Sigma *Escherichia coli* strain O55:B5) was dissolved in water and applied to cells in culture media at a final concentration of 10 μg/mL (Boutros *et al.* 2002; Horn *et al.* 2010). Cells were incubated with LPS in the culture media for 30 min, 1 hr, or 2 hr before harvesting by scraping. Total RNA was isolated using a QIAGEN RNeasy kit, with final elution in water. RNA quality and concentration were assessed using a Nanodrop 8000 spectrophotometer (Thermo-Scientific). cDNA synthesis was performed as outlined previously. Gene expression levels were assessed by qPCR in triplicate and normalized to *alphaTub84B* and *Gapdh1*. Fold induction was determined by comparing expression levels in treated cells *vs.* non-treated cells. qPCR reactions included each primer at 0.1 μM in an 11 microliter reaction volume.

### Online implementation of FlyPrimerBank

The user interface was implemented as a collection of CGI scripts written in Perl. They are hosted on a shared server provided by the Research IT Group (RITG) at Harvard Medical School. The database is hosted on a MySQL server also provided by RITG. The alignment of the virtual PCR product on the target transcript is drawn on an HTML5 canvas using JavaScript.

## Results

### Primer design and annotation

qPCR has become commonplace for transcript abundance analysis. Typical applications involve monitoring the amplification products indirectly, such as by measuring binding-dependent fluorescence of specific dyes (*e.g.*, SYBR Green). The technique, however, is vulnerable to nondesirable side products such as primer dimers or mispriming to nontarget sites, a significant concern when a sample contains thousands of transcripts. Most existing primer design programs are predicted based solely on the target sequence. By contrast, the PrimerBank algorithm takes into account the complexity of entire transcriptomes and applies stringent primer cross-reactivity filters in addition to running the NCBI Basic Local Alignment Search Tool (BLAST) (Wang and Seed 2003). The algorithm used for PrimerBank primer design was proven optimal for real time PCR analysis of gene expression, and primers were experimentally evaluated. It was shown to achieve 94% success in terms of gene amplification specificity as assessed by thermal analysis and approximately 82% confirmed with respect to DNA sequence identity, assessed by sequencing PCR products following gel electrophoresis (Spandidos *et al.* 2010; Wang and Seed 2003; Wang *et al.* 2012). Thus, to begin to create a comprehensive qPCR primer resource for *Drosophila*, we first chose to use the PrimerBank algorithm to design up to three primer pairs targeting the CDS region for all *Drosophila* protein-coding genes (FlyBase release 5.44). By design, all of these primer sets are predicted to be gene-specific and isoform non-specific. Our decision to target CDS regions is based on the observation that annotations of untranslated regions (UTRs) change much more frequently than CDS regions. We compared transcript annotations from FlyBase release 5.34 (February 18, 2011) with those from FlyBase release 5.44 (March 2, 2012). We found that 833 transcripts were removed, 3407 new transcripts were added, and 2917 transcripts have different sequences. The sequence changes of the 2917 transcripts occurred only in the UTR regions (Hu *et al.* 2013).

**Figure 1:**
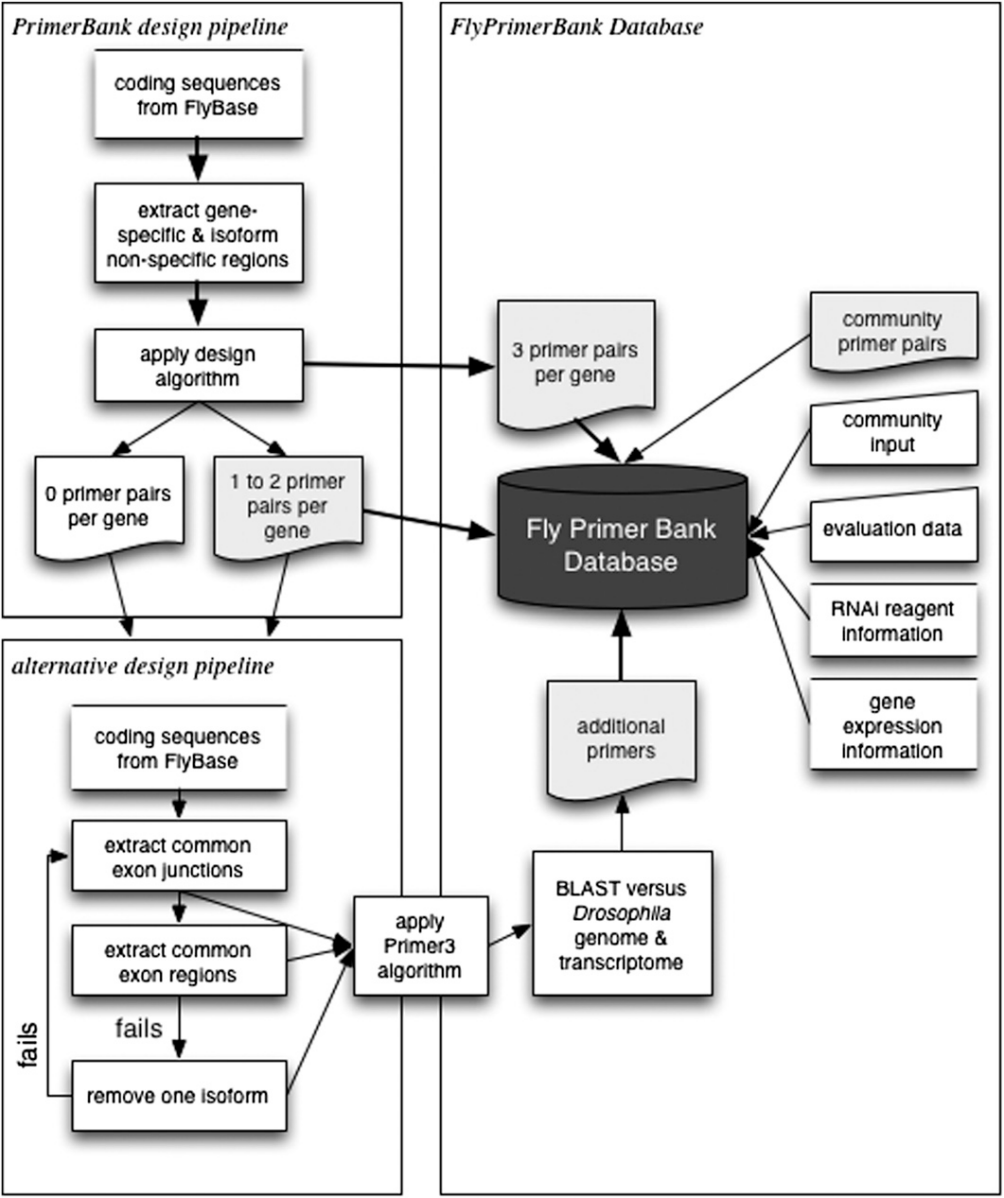
Primer design and annotation pipeline.

Using the PrimerBank algorithm, three primer pairs were successfully designed for 11,688 fly protein-coding genes. Primers were selected based on stringent criteria, in particular with regard to the sequence specificity check step (details described in Wang and Seed 2003). A total of 574 genes failed the design process completely and 1647 genes are covered with only 1 or 2 primer pairs. To increase primer coverage, we implemented an alternative algorithm for these genes using Primer3 for primer design. Common exon-exon junction regions, as well as common exons shared by all isoforms, were extracted from FlyBase release files (r5.44) and used as the input for Primer3. For a small number of genes, no common region(s) could be identified. We therefore repeated the process, removing one isoform at a time, to identify common region(s) among the remaining isoforms. We make note of these exceptional cases and users are alerted when these genes are queried on the website. The primers designed using Primer3 were ranked based on predicted gene specificity and assessed using BLAST, searching *Drosophila* transcriptome and genome sequences. The top three designs were selected to supplement the database (Figure 1). By implementing the alternative algorithm Primer3, which is less stringent than PrimerBank in terms of primer specificity as well as coverage for all gene specific isoforms, we were able to design at least 3 pairs of primers for each *Drosophila* protein-coding gene.

In total, FlyPrimerBank contains 45,158 predesigned primer pairs for 13,860 *Drosophila* genes with at least three primer pairs per gene (Table 1). In total, 37,647 primer pairs were designed using PrimerBank and 7511 primer pairs were designed using the alternative approach. A total of 44,951 primer pairs (99.5%) are predicted to be gene specific and isoform nonspecific, whereas 207 primer pairs (0.5%) are predicted to amplify most but not all isoforms. In addition, 10,635 primer pairs (24%) are exon-junction spanning, whereas 34,523 primer pairs (76%) amplify regions within exons. The average predicted PCR product is between 50 and 250 bps, and the average theoretical melting temperature for any primer pair is approximately 60°. After primer design, we systematically evaluated the overlap of each primer with publicly available dsRNA reagents, which include the genome-wide dsRNA amplicon library for cell-based studies from the DRSC, as well as long dsRNA hairpins in transgenic fly stocks from NIG (NIG-FLY), VDRC, and Transgenic RNAi Project of Harvard Medical School (TRiP) (Dietzl *et al.* 2007; Flockhart *et al.* 2011; Mohr *et al.* 2010; Ni *et al.* 2009, 2011; Yamamoto 2010). Annotation of the overlap with RNAi reagents will help scientists choose primer pairs that avoid the reagent itself, making them suitable for confirmation of RNAi-based knockdown (Table 1).

**Table 1:** FlyPrimerBank statistics

All primers	45,158 (for 13,860 Genes), n (%)
Designed by PrimerBank algorithm	37,647 (83)
Designed by alternative algorithm	7511 (17)
Isoform nonspecific	44,951 (99.5)
Isoform specific	207 (0.5)
Exon-exon junction spanning	10,635 (24)
Nonexon-exon junction spanning	34,523 (76)
Ovelap with any DRSC RNAi reagents	17,325 (38)
Overlap with any NIG RNAi reagents	11,742 (26)
Overlap with any VDRC-GD RNAi reagents	9901 (22)
Overlap with any VDRC-KK RNAi reagents*^a^*	6591 (15)
Overlap with any TRiP long hairpin Reagents*^a^*	1520 (3)

a Please note that the percentage overlap is low for these in part because they are not full-genome collections.

### Evaluation of FlyPrimerBank

**Figure 2:**
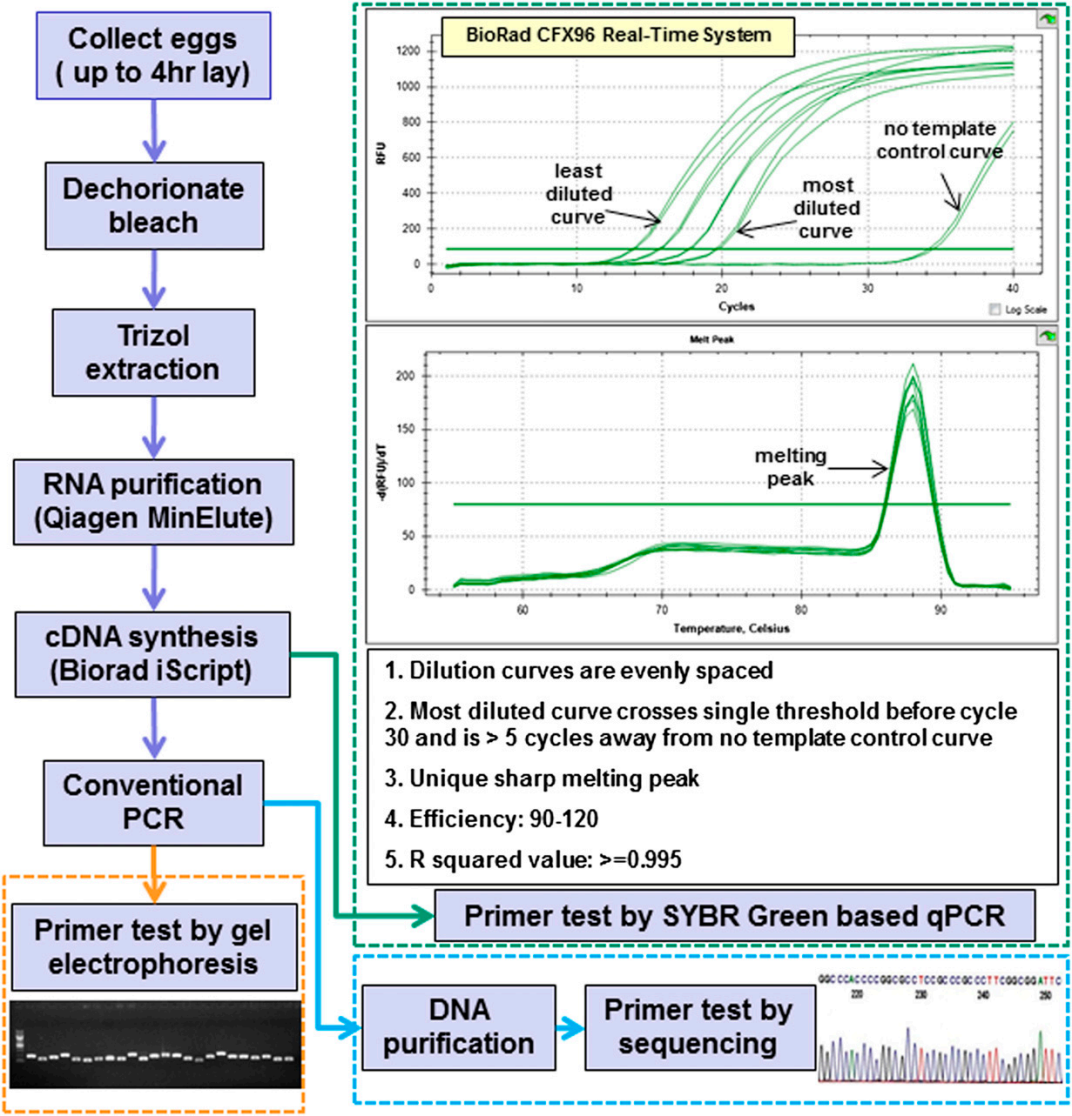
Primer testing pipeline and qPCR testing criteria.

We established a primer-testing pipeline using cDNA isolated from early *Drosophila* embryos in compliance with the “Minimum Information for the publication of real-time Quantitative PCR Experiments” (*i.e.*, MIQE) guidelines (bustin *et al.* 2009). Primers were tested by thermal analysis using SYBR Green-based qPCR as well as by size analysis using gel electrophoresis and sequencing after conventional PCR. To generate the cDNA template, embryos (0−4 hr) were collected and RNA was extracted, enriching for RNAs larger than 200 bps. Purified RNA was treated with DNAse and subsequently used for *in vitro* transcription to generate a cDNA library. cDNA was then serially diluted four times, starting with 1 μg of cDNA and decreasing the concentration with each dilution by a factor of four. R-squared values and primer efficiencies were calculated using Bio-Rad CFX Manager based on the results of a two-step qPCR program. Using preliminary data, we established acceptance criteria for assay performance (Figure 2), which included 90–120% PCR amplification efficiency, linear regression with R-squared values >0.995, and the following three visual features of amplification/melting calibration curves: (1) dilution curves are evenly distributed with two cycles separating each, indicative of a linear dynamic range; (2) the curve corresponding to the most diluted sample crosses a single threshold before cycle 30 and is at least five cycles away from a no template control (reaction mix and primers with no cDNA template); and (3) a single unique and sharp melting peak is observed (Supporting Information, Figure S1).

To establish a test set of genes, we considered genes for which there is evidence of expression in early embryos based on RNA-seq data (Graveley *et al.* 2011). To first determine a cutoff for “expressed” genes, we chose six genes of varied expression with reads per kilobase per million mapped reads values between 0 and 7 in 0- to 2- and 2- to 4-hr embryonic RNA-Seq datasets. Primer testing indicated that transcripts with reads per kilobase per million mapped reads values greater than 3 at any of the two time points were suitable for qPCR-based gene expression analysis (Figure S2). We next randomly selected one FlyPrimerBank primer pair for each of 326 randomly selected genes expressed in 0- to 4-hr-old *Drosophila* embryos, on the basis of the aforementioned criteria, for experimental qPCR primer testing. Our thermal analysis using cDNA isolated from 0- to 4-hr-old *Drosophila* embryos revealed that 86% of the primer pairs tested met our acceptance criteria (Table S1).

We also performed conventional PCR using the same cDNA as the template and separated the PCR products on an agarose gel to assess if single bands of the expected size were generated. All of the primer pairs in the 326-gene evaluation set generated PCR products with correct sizes as judged by gel electrophoresis. In addition, PCR products from conventional PCR were purified and sequenced using independently designed sequencing primers downstream of the forward qPCR primers (Figure 2). The sequencing validation rate varied from 71 to 98% and was highly correlated with the size of the PCR product (Figure S3). For example, 98% of PCR products longer than 150 bp were validated, and 80% of PCR products longer than 100 bp were validated. We suspect that the majority of sequence validation failures were caused by a technical limitation, *i.e.*, the difficulty of directly sequencing small PCR products based on the observation that low-quality reads were generated even after we optimized the PCR product purification step and repeated the sequencing a number of times.

**Figure 3:**
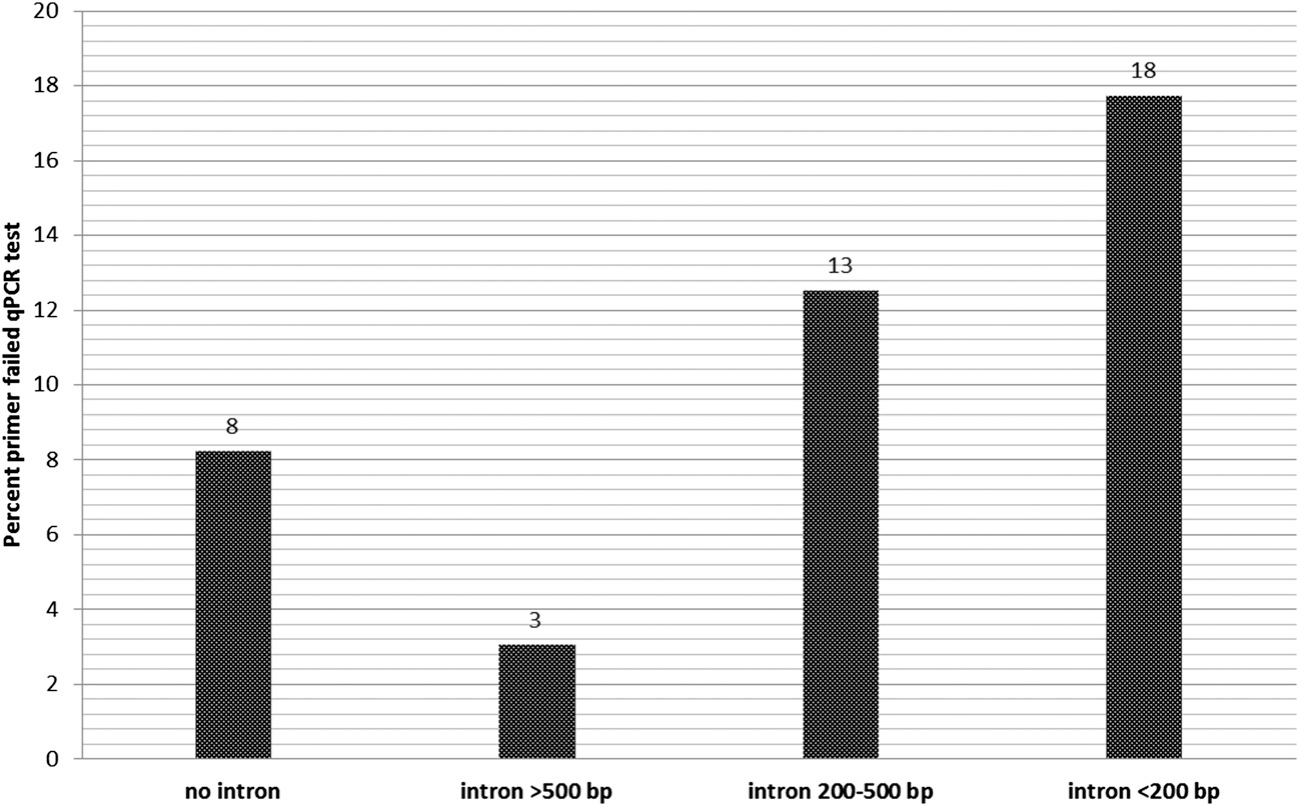
Primers that failed qPCR testing as a function of intron size.

We next asked whether primer failure correlated with any features of the PCR amplified regions. We found enrichment among “failed” primers for primers that span exon:exon junctions where the spliced intron is small (Figure 3). In addition, with a bigger dataset that includes results from additional studies (R. Sopko, personal communication), we found that primers corresponding to genes expressed at low levels as well as genes that are “poorly” understood (*i.e.*, genes associated with smaller numbers of Gene Ontology terms or publications) are more likely to fail than the primers targeting the genes expressed at high levels and/or relatively well-studied genes (*i.e.*, genes with more Gene Ontology terms and publications).

### Applications of FlyPrimerBank

#### Assessing gene knockdown efficiency in vivo:

**Figure 4:**
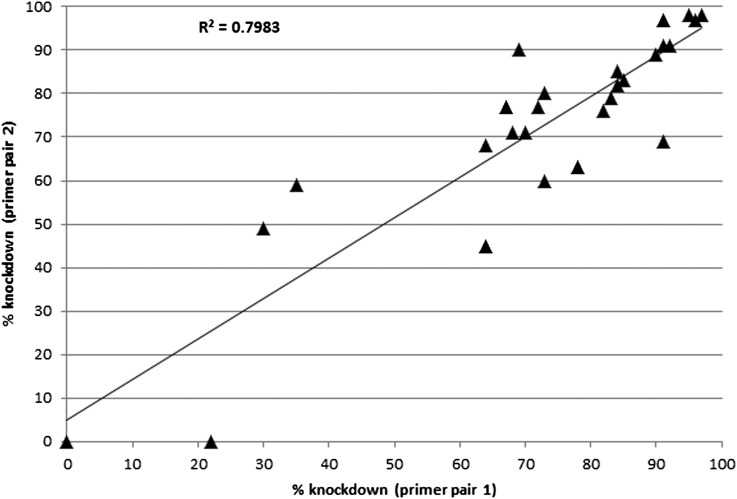
Observed RNAi knockdown levels are independent of primer design.

We next evaluated the utility of primers in FlyPrimerBank to assess knockdown in transgenic fly lines bearing shRNAs targeting embryonic *Drosophila* protein kinases and phosphatases (KPs). We first assembled a target list of KP genes. A few studies have been performed to identify *Drosophila* KPs on a genome-wide level using sequence comparison-based algorithms (Manning *et al.* 2002; Morrison *et al.* 2000). To supplement the gene list from these publications, we mined *Drosophila* structural and functional gene annotations from public databases. Additionally, we mapped human KP genes to fly genes using DIOPT, an ortholog prediction tool (Hu *et al.* 2011). The assembled KP list contains 268 kinase and 112 phosphatases (Table S2). Based on modEncode RNA-seq analysis (Graveley *et al.* 2011), we selected 474 TRiP fly stocks bearing shRNAs targeting 344 KPs that are expressed (FPKM > 3) in early *Drosophila* embryos for knockdown assessment. To measure transcripts in early embryos (0−4 hr) expressing a unique KP-targeting shRNA as compared to embryos expressing a control shRNA targeting EGFP, we established a medium-throughput pipeline again compliant with the MIQE guidelines (Bustin *et al.* 2009). For 27 lines, we assessed transcript levels using two different primer pairs. Linear regression of knockdown levels of this subset generated an R-squared value of 0.8 (Pearson correlation co-efficiency was 0.9; Figure 4), indicating that primers of independent designs led to similar conclusions regarding the level of knockdown. Furthermore, our analysis revealed that 60% of the transgenic lines tested achieved 60% or more down-regulation of their intended target transcript(s). Moreover, we found that in nearly all cases for which the shRNA was associated with an embryonic phenotype, we measured more than 60% knockdown of the intended transcript (Sopko, Foos, Binari, Perkins and Perrimon, unpublished data), further substantiating our primer design and qPCR testing approach. The lines that failed to achieve 60% knockdown are highly enriched for shRNAs targeting UTRs (*P* = 0.004), likely reflecting inaccuracies in UTR annotation (Hu *et al*. 2013).

#### Assessing transcriptional responses in cultured cells:

**Figure 5:**
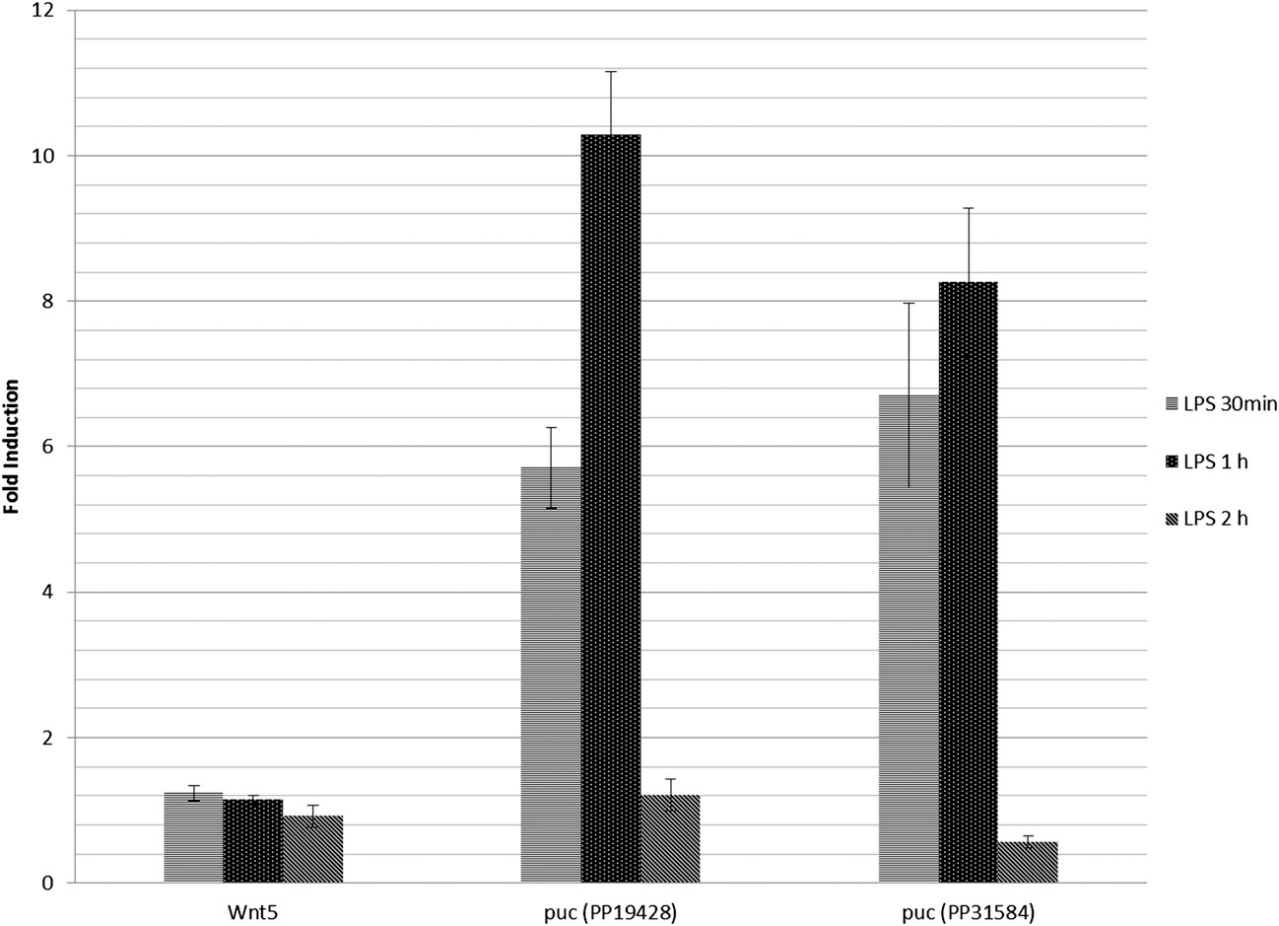
Monitoring JNK pathway activation. The *puc* gene is a direct target of the JNK pathway and is commonly used as a reporter for JNK activation (Boutros *et al.* 2002). A transient response of *puckered* (down after 2-hr stimulation) upon LPS stimulation in a *Drosophila* S2 cell line was observed. *Wnt5* serves as a negative control. Two independent *puckered* qPCR primer pairs (PP19428 and PP31584) in FlyPrimerBank show a similar trend in response.

We used primers in FlyPrimerBank to monitor activation of the JNK signaling pathway (Figure 5). Activation of the JNK pathway has been extensively studied in *Drosophila* cell culture (Bond *et al.* 2008; Park *et al.* 2004; Pereira *et al.* 2011; Stronach 2005). Various endogenous or immune stimuli can activate the pathway, including LPS, components of the cell wall of gram-negative bacteria. The *puckered* (*puc*) gene is a direct target of the JNK pathway and transcriptional up-regulation of *puc* is commonly used as a reporter of JNK activation. We assessed JNK pathway stimulation in *Drosophila* S2 cells by monitoring *puc* expression following the addition of LPS to culture media for 30 min, 1 hr, and 2 hr. Wnt5, a ligand of the Wnt/Wg signaling pathway, whose regulation is considered independent of JNK pathway activity in this cell line and expression level is comparable with *puc*, was used as a negative control. The transcriptional levels of *puc* and *Wnt5* were analyzed using SYBR Green-based qPCR analysis with primer pairs from FlyPrimerBank, validated using the same primer analysis procedure and acceptance criteria described previously. We observed similar levels of transient up-regulation of *puc* expression (down after a 2-hr stimulation) with two independent primer pairs. Meanwhile the transcript level of *Wnt5* remained stable during the time course. The rapid up-regulation of *puc* expression detected using our qPCR assay is characteristic of the LPS transcriptional response in the S2 Drosophila cell line (Park *et al.* 2004).

Our first application of FlyPrimerBank primers involved the evaluation of RNAi knockdown efficiency in early *Drosophila* embryos and the second examined transcriptional regulation by the JNK signaling pathway activity in cultured *Drosophila* cells. The two systems have completely distinct transcriptomes [*e.g.*, as shown by modEncode RNA-Seq (Cherbas *et al.* 2011; Graveley *et al.* 2011)], suggesting the utility of FlyPrimerBank primers for examination of transcript levels in diverse sample types (Figure S4).

### Implementation of a FlyPrimerBank online tool

**Figure 6:**
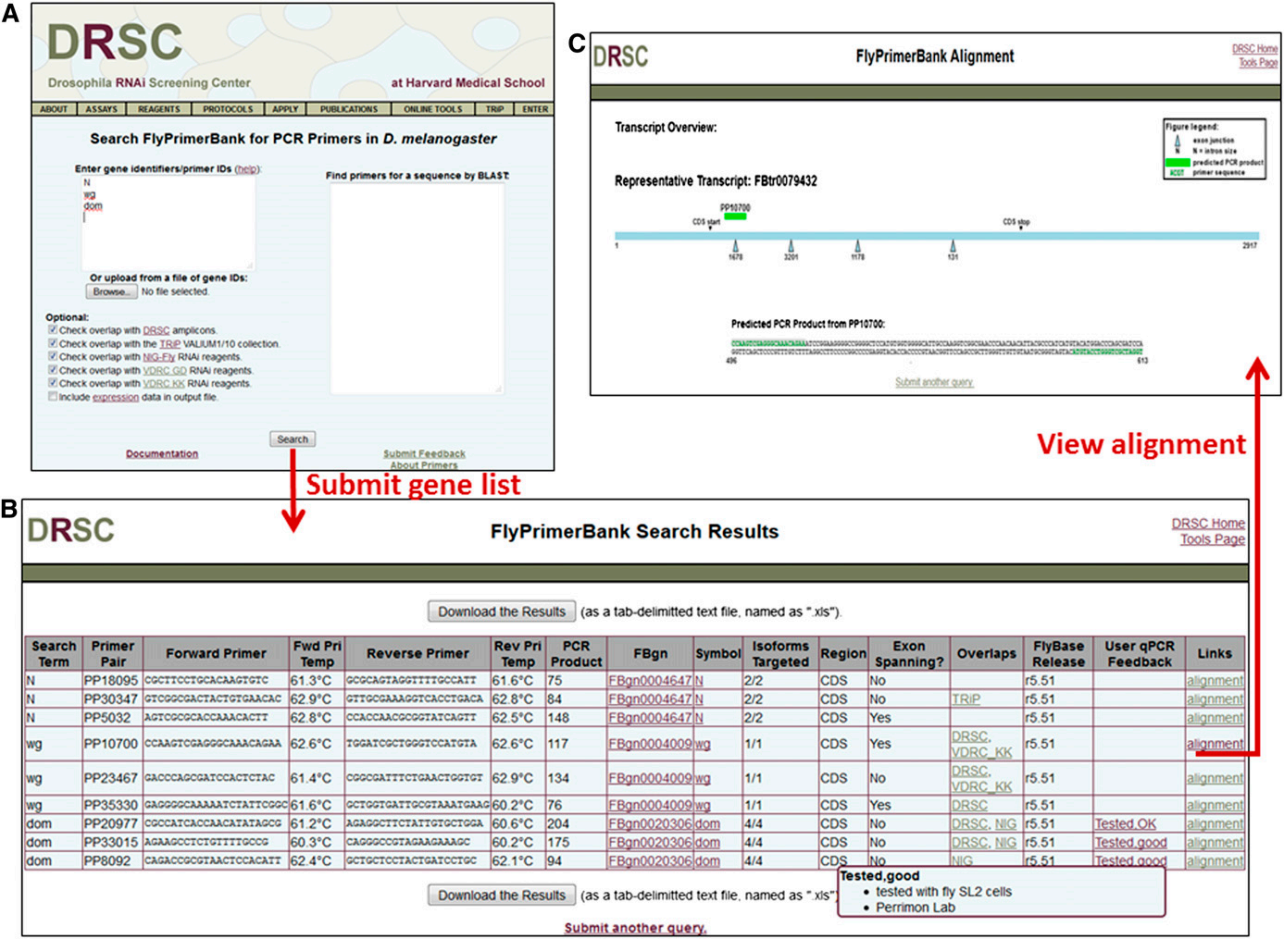
The FlyPrimerBank online user interface.

We implemented the FlyPrimerBank online tool to allow researchers to search for and identify qPCR primer pairs for *Drosophila* genes of interest. The user interface allows the user to search one or multiple genes with a single query (Figure 6). Gene ID mapping is built into the system so that users can query with any type of gene or protein identifier from FlyBase, including FBgn number, CG number, or gene symbol. The user interface also supports gene identification from sequence by BLAST search. The search results page shows the overlap of conceptually amplified products with RNAi reagents from various collections, as well as primer pair information, including sequences, melting temperatures, and the size of the predicted PCR product. Each primer pair is hyperlinked to a detailed annotation page, where PCR products are aligned to the reference transcript sequence, and exon-exon junctions and the CDS start and stop are visually displayed. As described previously, our results indicate that primer pairs are more likely to fail in instances in which the exon-exon junction they span has a small intron as compared to primer pairs spanning exon-exon junctions of large intron size or primers pairs that do not span exon-exon junctions. Therefore, on the detailed annotation page we also display the size of the intron spliced out at each exon-exon junction. Because of changes in gene annotations associated with new FlyBase releases, we plan to periodically re-annotate the primers. At the time of manuscript preparation, the annotation at FlyPrimerBank was based on FlyBase release 5.51 (May 7, 2013).

Unique to the FlyPrimerBank interface as compared to existing tools is a section where users can give feedback. Users can upload feedback about FlyPrimerBank primers they have tested and submit primers that were not designed by FlyPrimerBank but have been experimentally validated. This information will be displayed and as it accumulates, this added information will help researchers choose the best primer pairs, as well as allow us to identify genes for which new primers should be designed. Notably, we found that expression levels correlate with primer failure with respect to our thermal analysis criteria (Figure 2). However, a primer considered to have “failed” in our early embryo tests is not necessarily a suboptimal primer design because it might meet criteria when levels of the gene are higher (*e.g.*, at other developmental stages or in specific tissues). As such, users are required to submit information regarding the tissue, stage or cell type from which cDNA was isolated when submitting feedback on the results obtained with a given primer pair. To help assess potential qPCR targets and troubleshoot qPCR failure, we also provide users with gene expression data from a large-scale effort to determine expression levels in various cell lines and embryonic developmental stages (Cherbas *et al.* 2011; Graveley *et al.* 2011).

FlyPrimerBank is a comprehensive qPCR primer database for *Drosophila* that facilitates the selection of primers for small- or large-scale studies. FlyPrimerBank is more flexible than most resources in that it provides an interface for submission of user feedback on primers in the database and for submission of alternative experimentally validated qPCR primer sequences. Evaluation of RNAi reagents by qPCR is becoming common practice for gene loss-of-function studies. However, the long dsRNA reagents widely used for *Drosophila* genes often overlap regions amplified by qPCR primers, potentially confounding their evaluation. For this reason, our systematic qPCR primer design strategy considers long dsRNA reagents from several public resources and relevant information is made available online. The results of our quality analyses, from both *in vivo* and cell-based studies, demonstrate the utility and quality of the resource.

## Supplementary Material

Supporting Information
