# Supplementary material for: FlyPrimerBank: An Online Database for Drosophila melanogaster Gene Expression Analysis and Knockdown Evaluation of RNAi Reagents
Source: G3 (Bethesda). 2013 Sep 1;3(9):1607–16. doi: 10.1534/g3.113.007021 (PMC3755921; doi:10.1534/g3.113.007021)
Supplement: Supporting Information [file supp_3_9_1607__index.html]

FlyPrimerBank: An Online Database for Drosophila melanogaster Gene Expression Analysis and Knockdown Evaluation of RNAi Reagents — Supporting Information 

# FlyPrimerBank: An Online Database for *Drosophila melanogaster* Gene Expression Analysis and Knockdown Evaluation of RNAi Reagents

## Supporting Information for Hu *et al.*, 2013

**Files in this Data Supplement:**

- Supporting Information - (PDF, 811 KB)
- Figure S1 - Primer suitability evaluation (PDF, 87 KB)
- Figure S2 - Determining the expression cutoff for primer evaluation (PDF, 483 KB)
- Figure S3 - Sequence validation success is related to PCR product size (PDF, 76 KB)
- Figure S4 - Early embryos and S2 cells have different transcriptomes (PDF, 272 KB)
- Table S1 - Results of primer pair testing (.xlsx, 27 KB)
- Table S2 - Protein kinase and phosphatase list (.xlsx, 23 KB)
