# Supplementary material for: FlyPrimerBank: An Online Database for Drosophila melanogaster Gene Expression Analysis and Knockdown Evaluation of RNAi Reagents
Source: G3 (Bethesda). 2013 Sep 1;3(9):1607–16. doi: 10.1534/g3.113.007021 (PMC3755921; doi:10.1534/g3.113.007021)
Supplement: Supporting Information [file supp_g3.113.007021_007021SI.pdf]

G3·Genes | Genomes | Genetics

FlyPrimerBank: An Online Database for *Drosophila melanogaster* Gene Expression Analysis and Knockdown Evaluation of RNAi Reagents

Yanhui Hu[*,§,**], Richelle Sopko[*,**], Marianna Foos[*], Colleen Kelley[*], Ian Flockhart[*,§], Noemie Ammeux[*], Xiaowei Wang[†], Lizabeth Perkins[*,§], Norbert Perrimon[*,‡], Stephanie E. Mohr[*,§]

[*] Department of Genetics, Harvard Medical School, 77 Avenue Louis Pasteur, Boston, MA 02115, USA

[§] *Drosophila* RNAi Screening Center, Department of Genetics, Harvard Medical School, 77 Avenue Louis Pasteur, Boston, MA 02115, USA

[†] Departments of Radiation Oncology, Washington University School of Medicine, St. Louis, MO 63108, USA

[‡] Howard Hughes Medical Institute, 77 Avenue Louis Pasteur, Boston, MA 02115, USA

[**] Authors contributed equally to this work

DOI: 10.1534/g3.113.007021

A

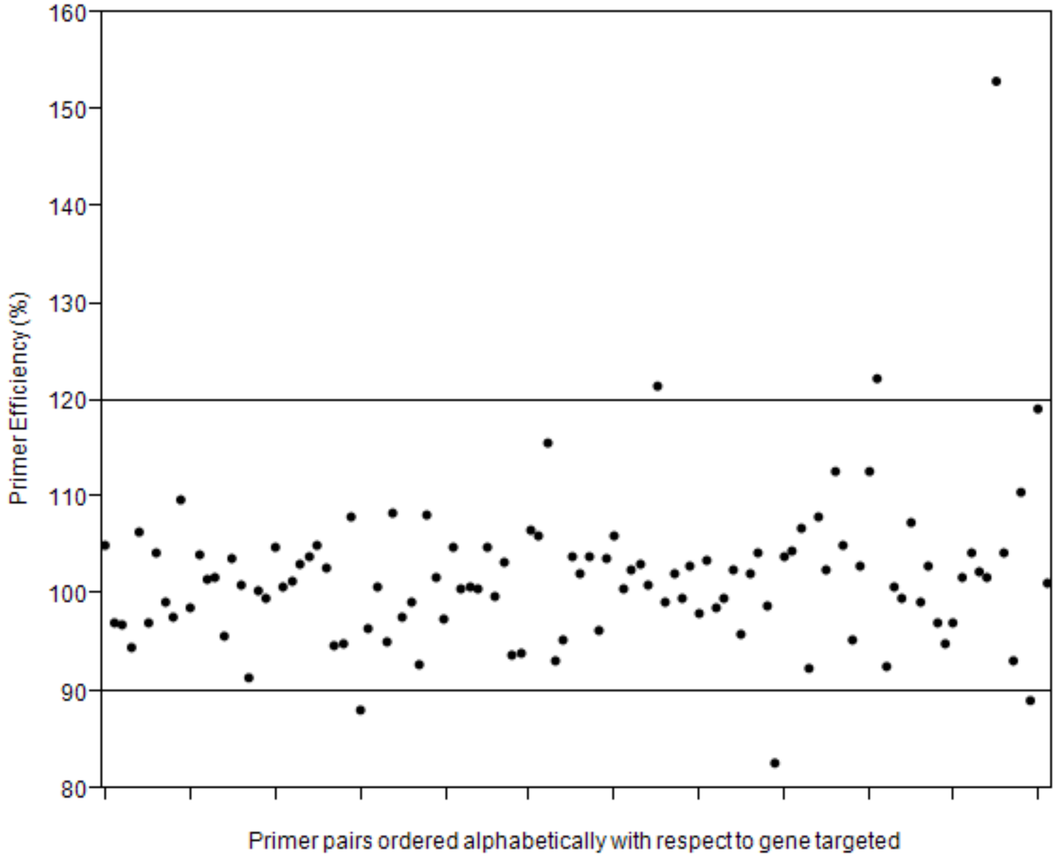
160
150
140
130
120
110
100
90
80
Primer Efficiency (%)
Primer pairs ordered alphabetically with respect to gene targeted

B

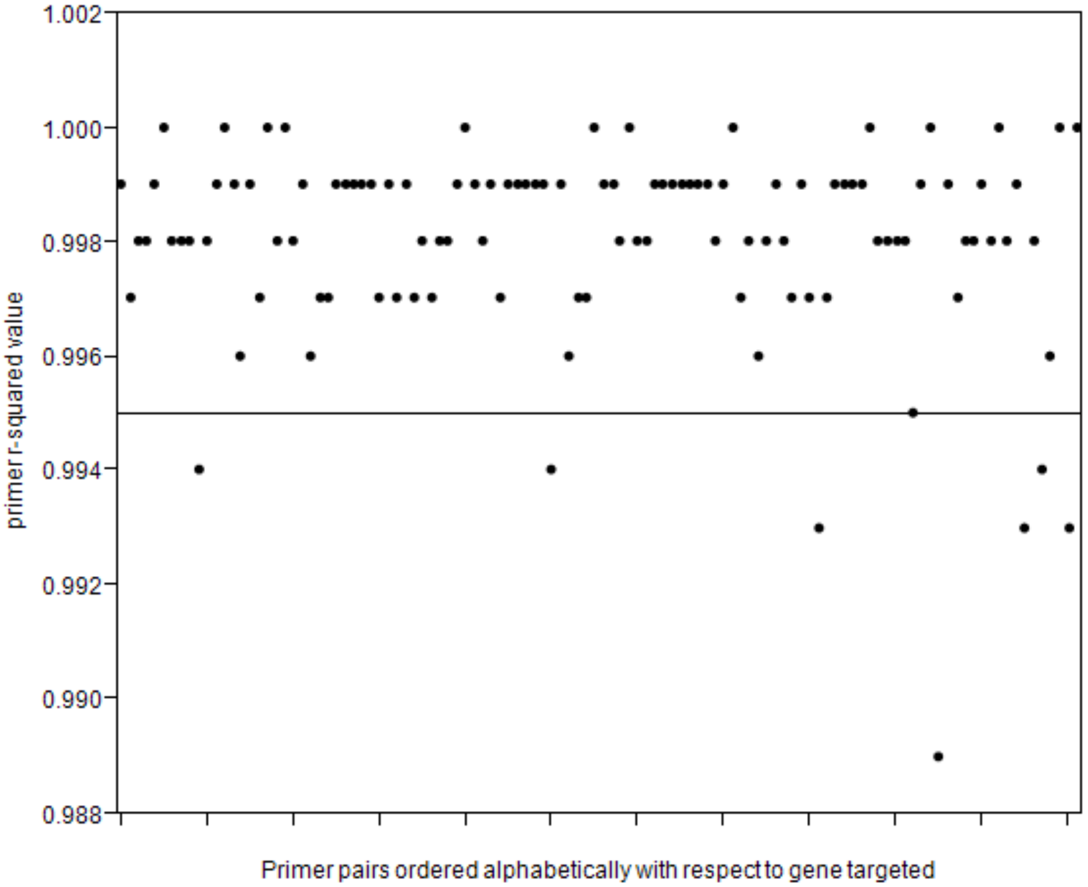
1.002
1.000
0.998
0.996
0.994
0.992
0.990
0.988
primer r-squared value
Primer pairs ordered alphabetically with respect to gene targeted

Figure S1 Primer suitability evaluation. (a) The upper and lower bounds for acceptable primer efficiency were 120% and 90%, respectively. Higher percentages suggest non-specific products, whereas lower percentages may reflect reaction inhibition. (b) Primers with an R-squared value (a measure of reproducibility) below 0.995 were considered unacceptable.

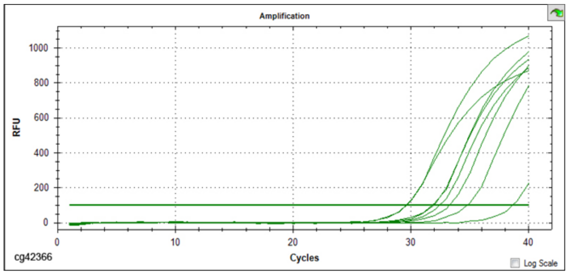

Primer for "CG42366"
RNASeq Data: 0 (0-2hr) 0 (2-4hr)

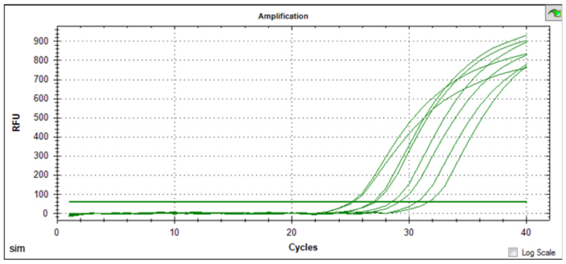

Primer for "sim"
RNASeq Data: 0 (0-2hr) 4 (2-4hr)

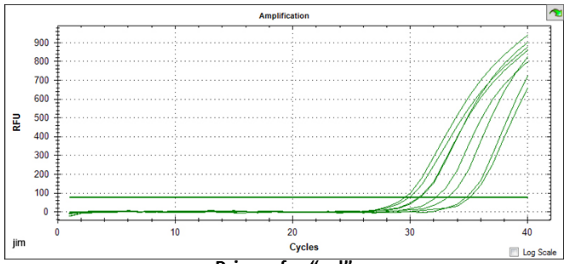

Primer for "pcl"
RNASeq Data: 0 (0-2hr) 0 (2-4hr)

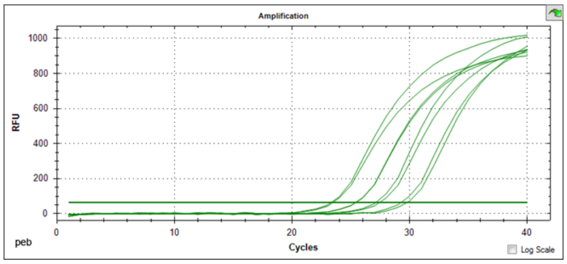

Primer for "peb"
RNASeq Data: 3 (0-2hr) 7 (2-4hr)

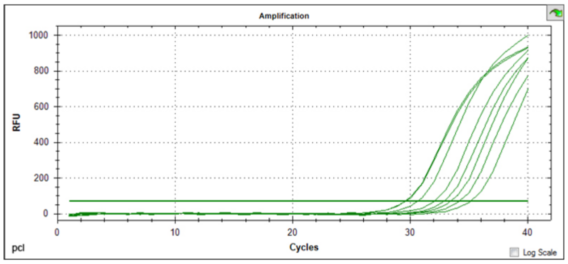

Primer for "jim"
RNASeq Data: 0 (0-2hr) 0 (2-4hr)

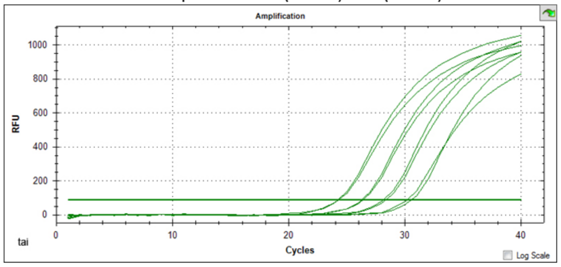

Primer for "tai"
RNASeq Data: 4 (0-2hr) 3 (2-4hr)

Figure S2 Determining the expression cutoff for primer evaluation. Genes represented in the left panels (*CG42866*, *pcl* and *jim*) do not express and are not suitable for primer evaluation in *Drosophila* early embryos while the genes in the right panels (*sim*, *peb* and *tai*) express and are suitable for primer evaluation.

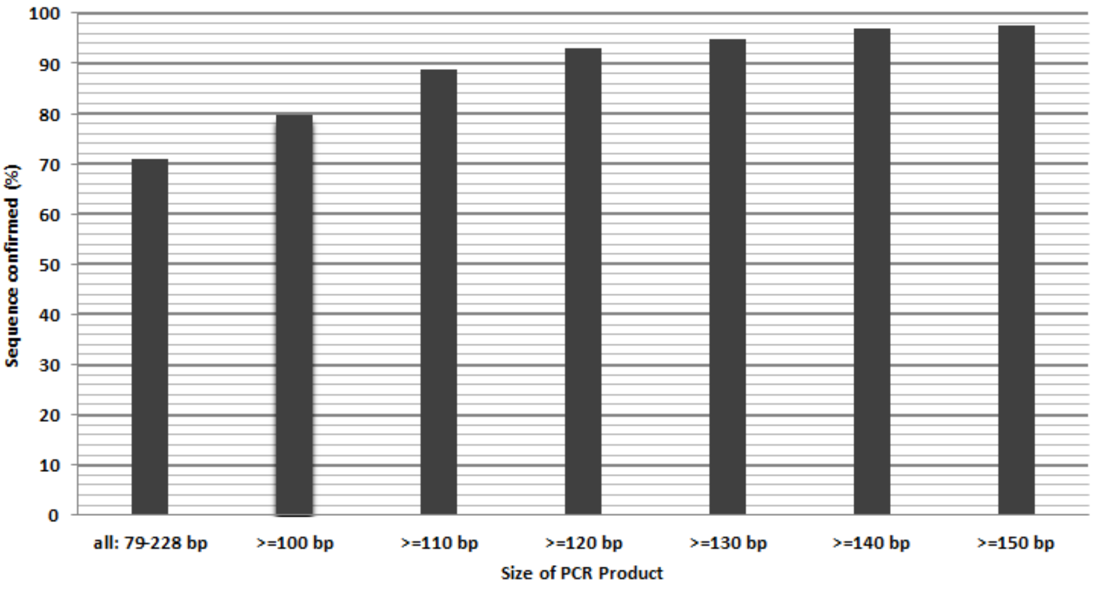

Figure S3 Sequence validation success is related to PCR product size.

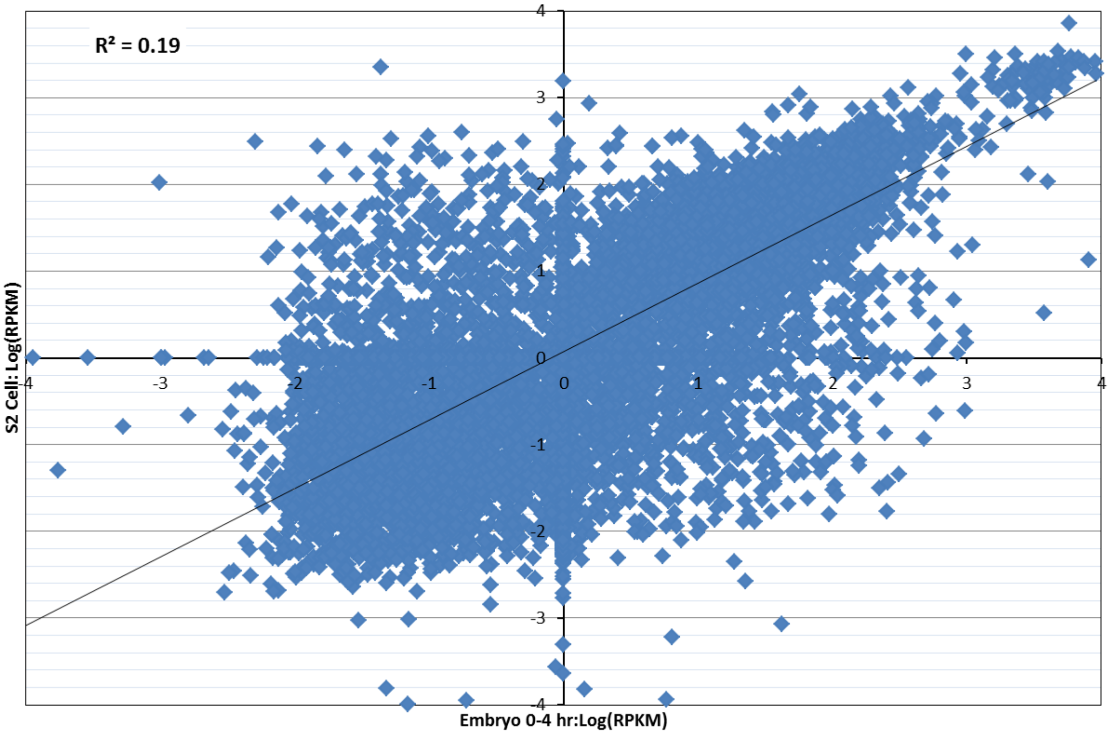

Figure S4 Early embryos and S2 cells have different transcriptomes. This graph is based on RNA-Seq data from the modEncode consortium.

Tables S1 and S2

Table S1: Results of primer pair testing.
Table S2: Protein kinase and phosphatase list

Available for download at http://www.g3journal.org/lookup/suppl/doi:10.1534/g3.113.007021/-/DC1.
