# Supplementary material for: FlyPrimerBank: An Online Database for Drosophila melanogaster Gene Expression Analysis and Knockdown Evaluation of RNAi Reagents
Source: G3 (Bethesda). 2013 Sep 1;3(9):1607–16. doi: 10.1534/g3.113.007021 (PMC3755921; doi:10.1534/g3.113.007021)
Supplement: Supporting Information [file supp_g3.113.007021_FigureS1.pdf]

A

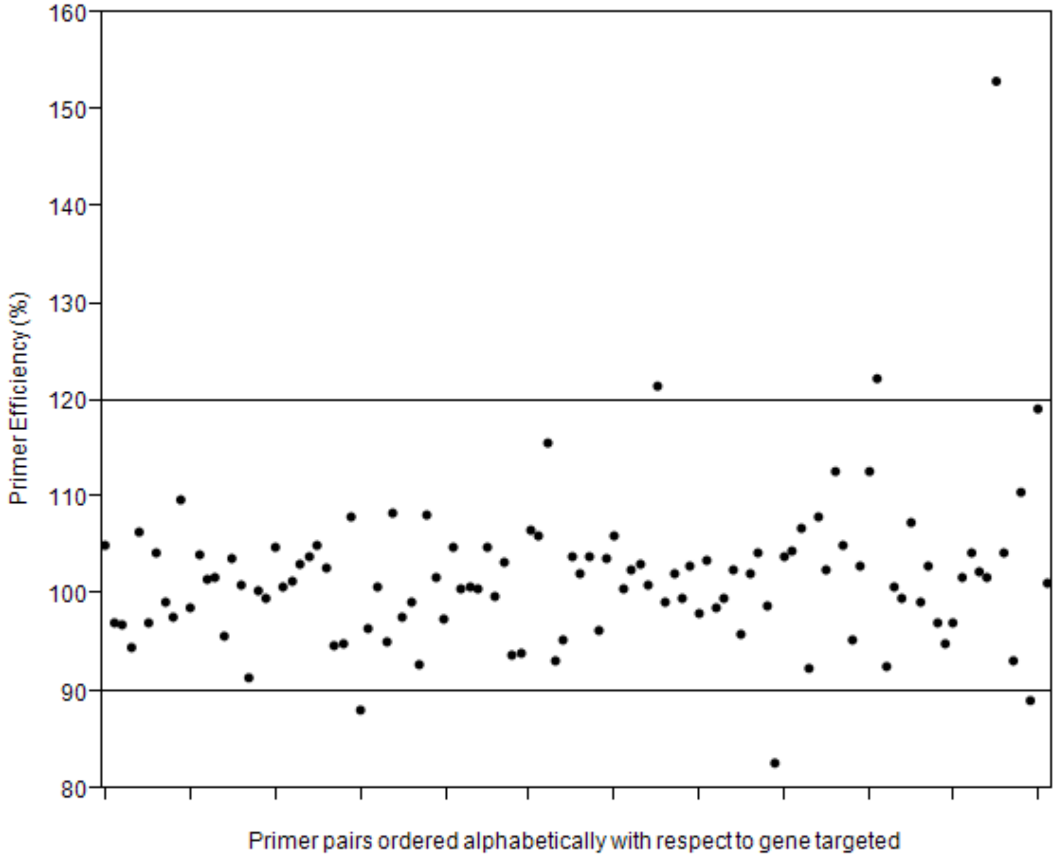
160
150
140
130
120
110
100
90
80
Primer Efficiency (%)
Primer pairs ordered alphabetically with respect to gene targeted

B

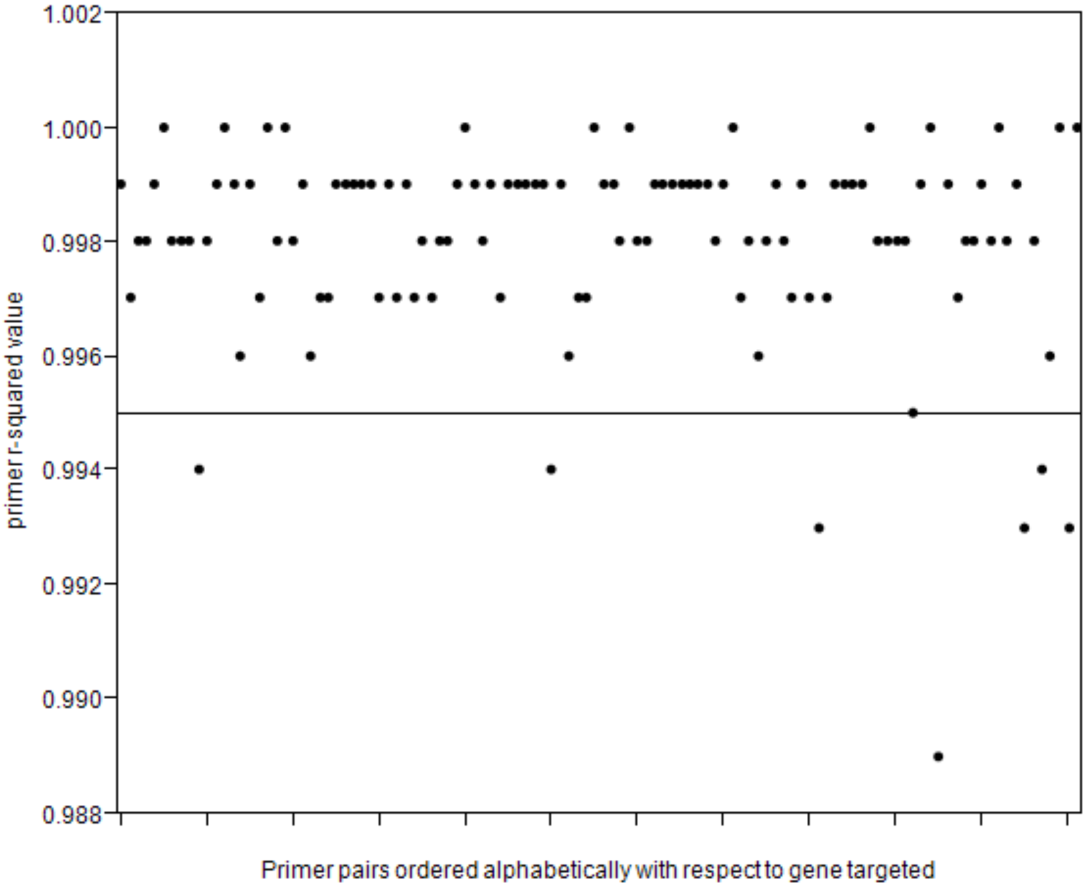
1.002
1.000
0.998
0.996
0.994
0.992
0.990
0.988
primer r-squared value
Primer pairs ordered alphabetically with respect to gene targeted

Figure S1 Primer suitability evaluation. (a) The upper and lower bounds for acceptable primer efficiency were 120% and 90%, respectively. Higher percentages suggest non-specific products, whereas lower percentages may reflect reaction inhibition. (b) Primers with an R-squared value (a measure of reproducibility) below 0.995 were considered unacceptable.
