# Supplementary material for: FlyPrimerBank: An Online Database for Drosophila melanogaster Gene Expression Analysis and Knockdown Evaluation of RNAi Reagents
Source: G3 (Bethesda). 2013 Sep 1;3(9):1607–16. doi: 10.1534/g3.113.007021 (PMC3755921; doi:10.1534/g3.113.007021)
Supplement: Supporting Information [file supp_g3.113.007021_FigureS2.pdf]

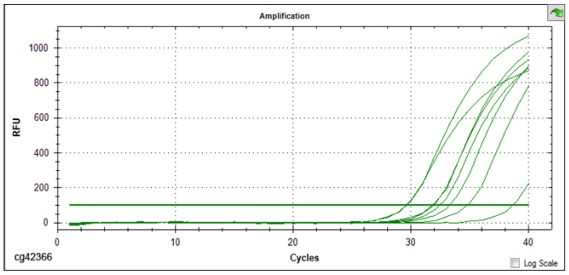

Primer for “CG42366”
RNASeq Data: 0 (0-2hr) 0 (2-4hr)

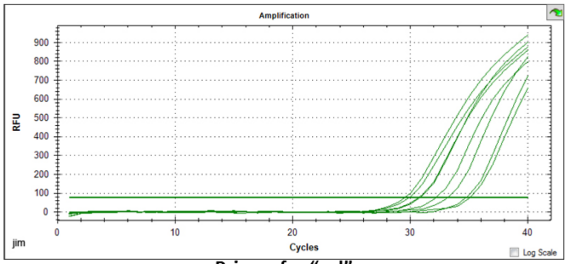

Primer for “pcl”
RNASeq Data: 0 (0-2hr) 0 (2-4hr)

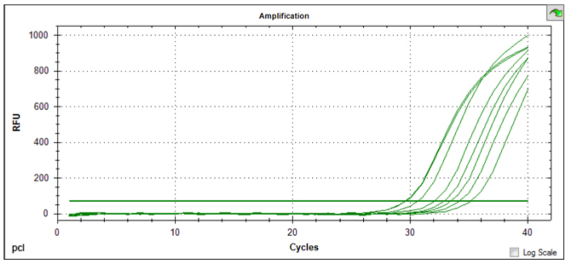

Primer for “jim”
RNASeq Data: 0 (0-2hr) 0 (2-4hr)

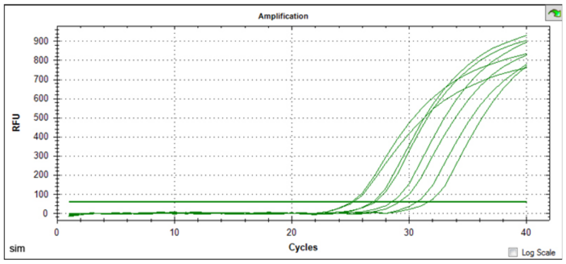

Primer for “sim”
RNASeq Data: 0 (0-2hr) 4 (2-4hr)

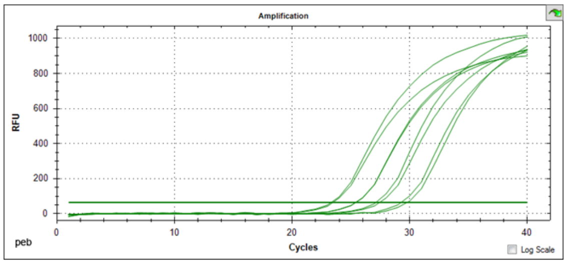

Primer for “peb”
RNASeq Data: 3 (0-2hr) 7 (2-4hr)

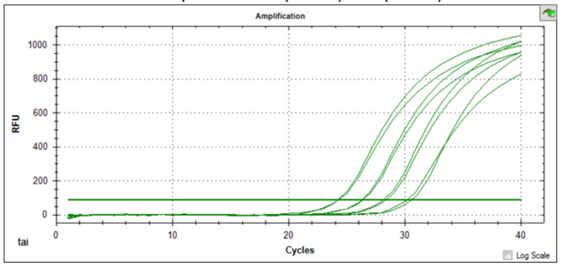

Primer for “tai”
RNASeq Data: 4 (0-2hr) 3 (2-4hr)

Figure S2 Determining the expression cutoff for primer evaluation. Genes represented in the left panels (*CG42866*, *pcl* and *jim*) do not express and are not suitable for primer evaluation in *Drosophila* early embryos while the genes in the right panels (*sim*, *peb* and *tai*) express and are suitable for primer evaluation.
