# Supplementary material for: FlyPrimerBank: An Online Database for Drosophila melanogaster Gene Expression Analysis and Knockdown Evaluation of RNAi Reagents
Source: G3 (Bethesda). 2013 Sep 1;3(9):1607–16. doi: 10.1534/g3.113.007021 (PMC3755921; doi:10.1534/g3.113.007021)
Supplement: Supporting Information [file supp_g3.113.007021_FigureS4.pdf]

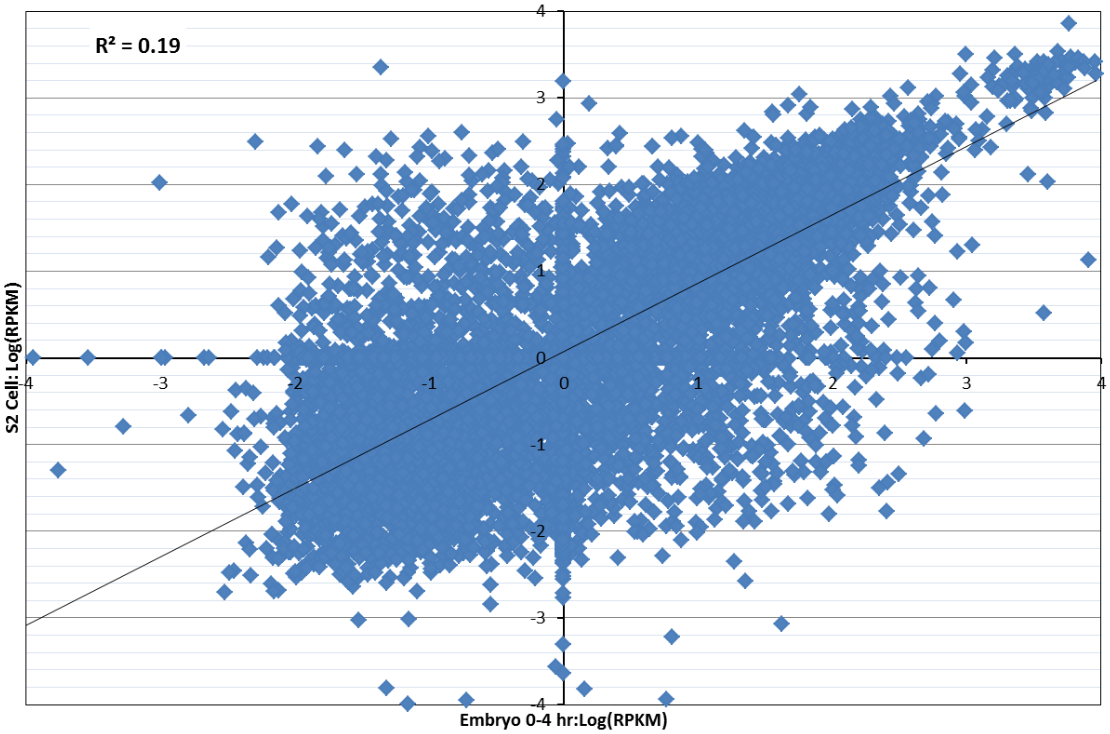

Figure S4 Early embryos and S2 cells have different transcriptomes. This graph is based on RNA-Seq data from the modEncode consortium.
